# Adverse cardiac effects of exogenous angiotensin 1-7 in rats with subtotal nephrectomy are prevented by ACE inhibition

**DOI:** 10.1371/journal.pone.0171975

**Published:** 2017-02-13

**Authors:** Louise M. Burrell, Daniel Gayed, Karen Griggs, Sheila K. Patel, Elena Velkoska

**Affiliations:** Department of Medicine, The University of Melbourne, Austin Health, Heidelberg, Victoria, Australia; Max Delbruck Centrum fur Molekulare Medizin Berlin Buch, GERMANY

## Abstract

We previously reported that exogenous angiotensin (Ang) 1–7 has adverse cardiac effects in experimental kidney failure due to its action to increase cardiac angiotensin converting enzyme (ACE) activity. This study investigated if the addition of an ACE inhibitor (ACEi) to Ang 1–7 infusion would unmask any beneficial effects of Ang 1–7 on the heart in experimental kidney failure. Male Sprague–Dawley rats underwent subtotal nephrectomy (STNx) and were treated with vehicle, the ACEi ramipril (oral 1mg/kg/day), Ang 1–7 (subcutaneous 24 μg/kg/h) or dual therapy (all groups, n = 12). A control group (n = 10) of sham-operated rats were also studied. STNx led to hypertension, renal impairment, cardiac hypertrophy and fibrosis, and increased both left ventricular ACE2 activity and ACE binding. STNx was not associated with changes in plasma levels of ACE, ACE2 or angiotensin peptides. Ramipril reduced blood pressure, improved cardiac hypertrophy and fibrosis and inhibited cardiac ACE. Ang 1–7 infusion increased blood pressure, cardiac interstitial fibrosis and cardiac ACE binding compared to untreated STNx rats. Although in STNx rats, the addition of ACEi to Ang 1–7 prevented any deleterious cardiac effects of Ang 1–7, a limitation of the study is that the large increase in plasma Ang 1–7 with ramipril may have masked any effect of infused Ang 1–7.

## Introduction

Activation of the renin-angiotensin system (RAS) [1] is recognised as a key pathogenic factor in the development of kidney disease and its cardiovascular complications. Within the RAS, angiotensin converting enzyme (ACE) converts angiotensin (Ang) I to the pro-fibrotic peptide Ang II, which mediates its effects via the Ang type 1 receptor (AT_1_R). Drugs in clinical use such as the ACE inhibitors (ACEi) and AT_1_R blockers slow but do not halt the rate of kidney and cardiac disease progression, prompting the search for new approaches.

New components of the RAS have been described including angiotensin converting enzyme 2 (ACE2) [2, 3] and Ang 1–7 [4] and are thought to play an important role in counter-regulating the adverse consequences of an activated RAS [5]. Ang 1–7 is produced following cleavage of Ang II by ACE2 [2, 3, 5] and exerts its effects via the *mas* receptor [6]. Exogenous administration of Ang 1–7 has cardio-protective and anti-fibrotic actions in experimental models of cardiovascular disease including myocardial infarction [7], hypertension [8], atrial fibrillation [9] and atherosclerosis [10]. In a mouse model of type 2 diabetes, Ang 1–7 prevents heart and lung dysfunction [11, 12], and prevents systemic hypertension, reduces renal fibrosis and normalises expression of renal RAS components [13].

However we and others have reported that in the presence of kidney disease, Ang 1–7 has deleterious rather than protective effects on the heart and kidney [14, 15]. Ang 1–7 infusion accelerates kidney injury in experimental type 1 diabetes [14], worsens kidney damage following unilateral ureteral obstruction (UUO) [16, 17], and has no beneficial renal effects in a model of focal segmental glomerulosclerosis [18]. In uninephrectomised sheep, renal responses to Ang 1–7 are altered resulting in vasoconstriction and sodium retension, effects that were reversed by AT_1_R blockade [19]. We have previously reported that Ang 1–7 infusion increases blood pressure, left ventricular hypertrophy (LVH) and fibrosis in rats with subtotal nephrectomy (STNx), and indirectly increases cardiac ACE activity [15]. We speculated that increased cardiac ACE not only increases the degradation of cardiac Ang 1–7, but also generates more of the profibrotic peptide Ang II, which may be the cause of the observed adverse effects [15]. Therefore, in the current study, we tested the hypothesis that the addition of ACE inhibition to exogenous Ang 1–7 may unmask beneficial cardiac effects of Ang 1–7 in kidney disease.

This study examined the effect of combining ACEi and exogenous Ang 1–7 on blood pressure, cardiac structure/function and plasma and cardiac tissue RAS components in a rat model of STNx, and determined if combination therapy would have additional benefits compared to ACEi alone.

## Materials and methods

### Experimental protocol

Experimental procedures were performed in accordance with the National Health and Medical Research Council of Australia guidelines for animal experimentation and were approved by the Animal Ethics Committee, Austin Health. Male Sprague Dawley rats (200-250g) were housed in a 12:12h light-dark cycle, with *ad libitum* food containing 0.4–0.6% NaCl (Norco) and water. STNx (n = 48) or sham surgery (n = 10) was performed in rats by right nephrectomy, and ligation of all but one of the extrarenal branches of the left renal artery as described previously [15, 20–24]. Animals received a dose of analgesic (buprenorphine, 20 μg/kg) following the procedure and were monitored daily for the length of the experiment. No adverse events were observed. STNx rats were randomly allocated to 10 days treatment with vehicle (0.9% saline, n = 12), the ACEi ramipril (oral, 1 mg/kg/day, n = 12), Ang 1–7 (s.c. 24 μg/kg/h, n = 12) via osmotic minipump (Model # 2002, Alzet, Cupertino, CA, USA), or combination (ramipril and Ang 1–7). Sham operated rats (Control) received vehicle.

On day 9, rats were housed in metabolic cages, and a urine sample collected for the measurement of creatinine. On day 10, rats were anaesthetised with intraperitoneal (i.p.) sodium pentobarbitone (60 mg/kg/body weight), and cardiac hemodynamics were determined using a micro-tipped pressure transducer catheter (Millar, 1.5F) inserted into the left carotid artery and advanced into the LV [15, 23, 24]. Data were analysed using Millar conductance data acquisition and analysis software, and systolic blood pressure, maximal rate of ventricular contraction (+dP/dt), and left ventricular end diastolic pressure (LVEDP) determined. Diastolic function was assessed by measuring the time constant of isovolumic relaxation (Tau), which assess active relaxation with higher values of Tau implying impaired relaxation [25].

Rats were then killed by a lethal dose of sodium pentobarbitone, decapitated and trunk blood collected into either lithium heparin tubes or into EDTA tubes containing 20μl/ml of blood of an inhibitor cocktail (50mM Na_2_EDTA, 0.2M N-ethylmaleimide and 1–2 TIU/mL aprotinin made up in saline). After centrifugation plasma was snap-frozen and stored at -80^°^C. The remnant kidney and heart were removed and weighed. The left ventricle (LV) was transversely dissected into 3 pieces, and one piece fixed in 4% paraformaldehyde and embedded in paraffin for histopathology. The remainder was snap frozen in isopentane and stored at -80°C for *in vitro* autoradiographic studies, and ACE2 activity assay.

### Drugs

Sodium pentobarbitone was obtained from Boehringer Ingelheim, Artarmon, NSW, Australia), Ang 1–7 from Auspep, Parkville, VIC, Australia, ramipril from sanofi-aventis, Germany.

### Biochemical analysis

Plasma creatinine was measured using an autoanalyser (Beckman Instruments, Palo Alta, CA, USA).

### Determination of cardiac collagen

Cardiac (LV) paraffin sections 4μm thick were deparaffinized, rehydrated, and then stained with 0.1% Sirius Red (Polysciences Inc) in saturated picric acid (picrosirius red) for 1 hour, differentiated in 0.01% HCl for 30 seconds, and rapidly dehydrated. Interstitial collagen volume fraction was determined by measuring the area of stained tissue within a given field, excluding vessels, artefacts, minor scars or incomplete tissue fields; 15–20 fields were analysed per animal. To measure perivascular collagen, all arteries in the LV section were analysed, and the whole artery including the adventitia was selected for assessment. For both interstitial and perivascular collagen, the area stained was then calculated as a percentage of the total area within a given field [26–28].

### Plasma angiotensin peptides

Blood for the measurement of angiotensin peptides was collected and stored as described above. The radioimmunoassays for Ang II and Ang 1–7 have been previously described [21, 29], and use antibodies for Ang II and Ang 1–7 raised in rabbit and guinea pig respectively, and the specific radio-isotopes, ^125^I-Ang II and ^125^I-Ang 1–7 made by Prosearch (Melbourne, Australia). The intra- and inter-assay coefficients of variation were 7.6% and 8.3% for Ang II and 4.5% and 10% for Ang 1–7.

### Plasma ACE activity

Blood collected in heparinised tubes was centrifuged, stored at 4^°^C and plasma ACE activity measured using an enzymatic assay as previously published [15, 30]. Briefly 5μl of plasma was incubated at 37^°^C with the ACE substrate hippuryl-His-Leu (1mM) in a total volume of 50μl buffer (0.4M sodium borate buffer, 0.3M NaCl, pH 8.3) in the presence and absence of EDTA (10μM) for 30 min. The rate of substrate cleavage was determined by comparison to a standard curve of the product His-Leu and expressed as nmole of substrate/ml of plasma/hr.

### *In vitro* autoradiography for cardiac ACE binding

Cardiac ACE was assessed by *in vitro* autoradiography on LV sections (20μm) in 10 rats/group using the specific radioligand ^125^I-MK351A (K_i_ = 30pmol/l) as previously described [20, 27]. Quantitation of ACE binding density in two LV sections from each animal (N = 7-8/group) was performed using a microcomputer imaging device (Imaging Research, Ontario, Canada), which measures the relative optical density of the radioactive labelling. Results are expressed as a percentage of binding in control rats.

### Plasma and cardiac ACE2 activity

LV membranes were prepared as described previously [20, 27] and 100μg of protein was incubated in duplicate with an ACE2 quenched fluorescent substrate (QFS), [(7-methoxycoumarin-4-yl)-acetyl-Ala-Pro-Lys (2, 4-dintirophenyl); Auspep, Parkville, Victoria, Australia], 10μM Z-Pro-prolinal (Auspep, Parkville, Victoria, Australia), with or without 100μM EDTA in a total volume of 200μl [15, 20, 22, 24]. The rate of substrate cleavage was determined by comparison to a standard curve of the free fluorophore, 4-amino-methoxycoumarin (MCA; Sigma, MO, USA) and expressed as nmole of substrate cleaved/mg of protein/hr. For plasma ACE2 activity, blood collected into heparinised tubes was centrifuged at 4°C and assayed as above. Results are expressed as nmole of substrate/ml of plasma/hr.

### Statistical analysis

Data are presented as mean ± standard error of mean (SEM). P values were calculated using a Student’s unpaired t-test when comparing Control vs. STNx and ANOVA followed by post-hoc Bonferroni analysis when comparing STNx+Veh to the treatment groups. For data with unequal variance, results were log-transformed and analysed using Kruskal-Wallis test with Dunn’s multiple comparison test. Significant differences were obtained when P<0.05.

## Results

### STNx and renal function

Table 1 shows the changes in physiological and biochemical parameters after STNx and the effect of intervention. Following STNx, rats had poor weight gain (*P<*0.01) and hypertrophy of the remnant kidney (*P<*0.001). Renal impairment was present as indicated by elevated plasma creatinine (*P<*0.001) compared with Control rats. Combination therapy reduced renal hypertrophy but had no effect on plasma creatinine. Neither ramipril nor Ang 1–7 affected renal parameters.

**Table 1:** Body weight, renal parameters and plasma RAS components.

	Control	Subtotal nephrectomy			
	Vehicle	Vehicle	Ramipril (1 mg/kg/day)	Ang 1–7 (24 μg/kg/h)	Ram +Ang 1–7
	(n = 10)	(n = 12)	(n = 12)	(n = 12)	(n = 12)
Body weight (g)	277 ± 4	209 ± 10***	230 ± 6	215 ± 5	235 ± 4
**Renal Parameters**					
Left kidney / body weight (g/100g)	0.39 ± 0.02	0.55 ± 0.03***	0.49 ± 0.01	0.50 ± 0.02	0.45 ± 0.01^##^
Plasma creatinine (mol/L)	20.6 ± 0.4	52.5 ± 0.5***	50.2 ± 0.5	46.1 ± 0.5	47.8 ± 0.4
**Plasma RAS components**					
ACE (nmol/ml/hr)	720.4 ± 86.5	673.2 ± 86.1	3.3 ± 2.2^###^	754.0 ± 98.5	1.8 ± 1.4^###^
ACE2 (nmol/ml/hr)	9.0 ± 0.6	8.9 ± 1.2	7.6 ± 0.9	9.6 ± 0.9	7.3 ± 0.3
Ang II (fmol/ml)	50.6 ± 11.9	51.6 ±14.5	85.9 ± 12.1	21.4 ± 2.9	110.7 ± 9.9^##^
Ang 1–7 (fmol/ml)	407.1 ± 64.0	316.6 ± 51.6	1335.0 ± 341.8^#^	258.7 ± 26.4	1572.0 ± 420.1^#^
Ang 1–7: Ang II ratio	12.3 ± 2.7	6.6 ± 0.7*	10.4 ± 0.9	11.4 ± 1.0^#^	8.4 ± 1.6

*P<0.05

***P<0.001 vs. control vehicle

#P<0.05

##P<0.01

###P<0.001 vs. vehicle treated STNx.

Data expressed as mean±SEM.

### Plasma RAS components

Table 1 shows the change in circulating RAS components after STNx and the effect of treatment. STNx was not associated with any change in plasma ACE and ACE2 activity or plasma Ang II and Ang 1–7 peptide levels compared with Control rats. The calculated Ang 1–7:Ang II ratio provides an index of the conversion of Ang II to Ang 1–7 and was significantly decreased (P<0.05) in STNx rats.

Both ramipril and combination therapy significantly reduced plasma ACE (P<0.001) and increased plasma Ang 1–7 (P<0.05). Combination therapy also increased plasma Ang II levels (P<0.01). Ang 1–7 infusion had no effect on circulating Ang II nor Ang 1–7 levels possibly due to its very short half-life (seconds) in rats [31], and as a result of increased breakdown due to elevated cardiac ACE activity. This is further supported by our previous findings that in control rats, Ang 1–7 infusion does increase plasma Ang 1–7 levels [15]. At the end of the study, all pumps had a minimal residual volume, and the concentration of Ang 1–7 in the residual volume was similar to that originally added to the pump (baseline Ang 1–7 level: 11.2 mg/ml; post-infusion residual volume Ang 1–7 level: 7.8 mg/ml).

### Blood pressure and cardiac function

**Fig 1:**
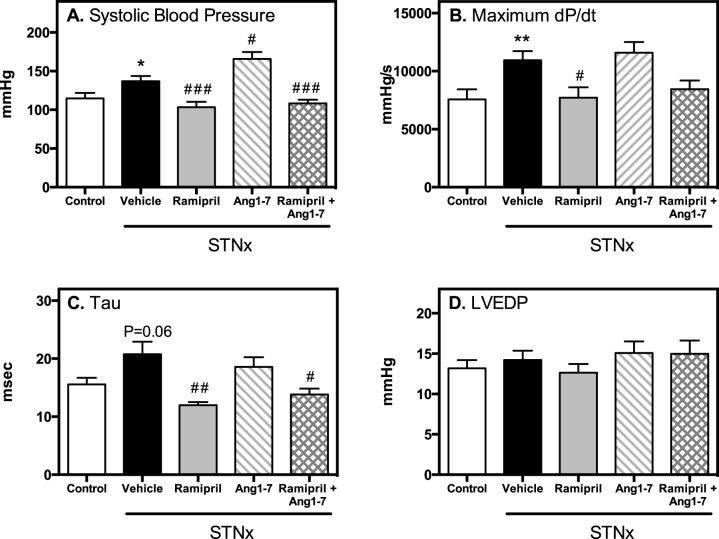
Blood pressure and cardiac function. Systolic blood pressure (A), ventricular contractility (B), time constant of isovolumic relaxation (Tau; C) and left ventricular end diastolic pressure (LVEDP; D) in control (n = 10) and STNx (vehicle, ramipril, Ang 1–7, ramipril + Ang 1–7; n = 12/group) rats. Data expressed as mean±SEM. *P<0.05, **P<0.01 vs. control and # P<0.05, ## P<0.01, ### P<0.001 vs. STNx

Fig 1 shows the change in blood pressure and cardiac function after STNx. Compared to control rats, STNx had elevated systolic blood pressure (Fig 1A, P<0.01), which was associated with increased left ventricular contractile force (Fig 1B, max. dP/dt, P<0.01). Diastolic dysfunction was also present, as shown by a trend for the time constant for isovolumic relaxation (Tau) to increase, which represents impaired active relaxation (Fig 1C, P = 0.06). There was no change in left ventricular end diastolic pressure (LVEDP) at this early stage of kidney impairment (Fig 1D).

Treatment with the ACEi ramipril decreased blood pressure (Fig 1A, P*<*0.01), reversed the hypercontractile state (Fig 1B, max. dP/dt P<0.05) and improved diastolic function (Fig 1C, Tau P<0.01). Ang1-7 infusion in STNx rats resulted in further increases in blood pressure (Fig 1A, P<0.05), with no change in other functional parameters. There were no additional benefits of combining ACEi with Ang 1–7 on cardiac parameters, but ramipril did prevent the increase in systolic blood pressure observed with Ang 1–7 alone.

### Cardiac hypertrophy and fibrosis

**Fig 2:**
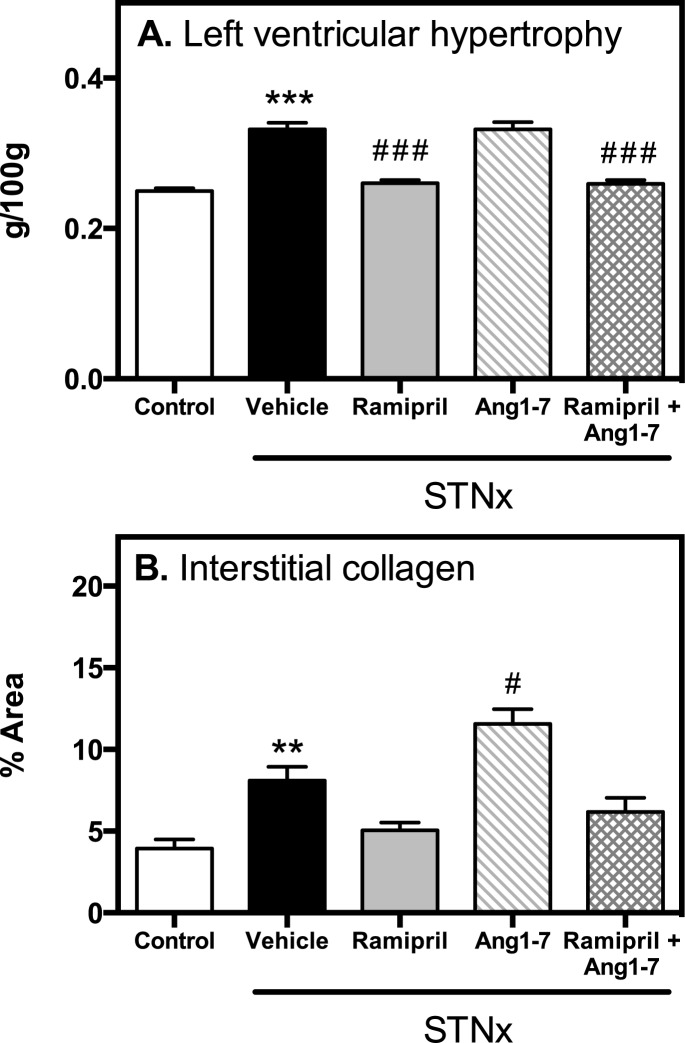
Cardiac hypertrophy and fibrosis. Left ventricular hypertrophy (A), interstitial collagen (B) in control (n = 10) and STNx (vehicle, ramipril, Ang 1–7, ramipril + Ang 1–7; n = 12/group) rats. Data expressed as mean±SEM. **P<0.01, ***P<0.001 vs. control and # P<0.05, ### P<0.001 vs. STNx

STNx was associated with LVH (Fig 2A, P<0.001) and interstitial fibrosis (Fig 2B, P<0.01). Ramipril alone and in combination with Ang 1–7 led to a significant decrease in LVH (Fig 2A, P<0.001). The increase in cardiac fibrosis associated with Ang 1–7 (Fig 2B, P<0.01) was prevented when used in combination with ramipril.

### Cardiac ACE and ACE2 activity

**Fig 3:**
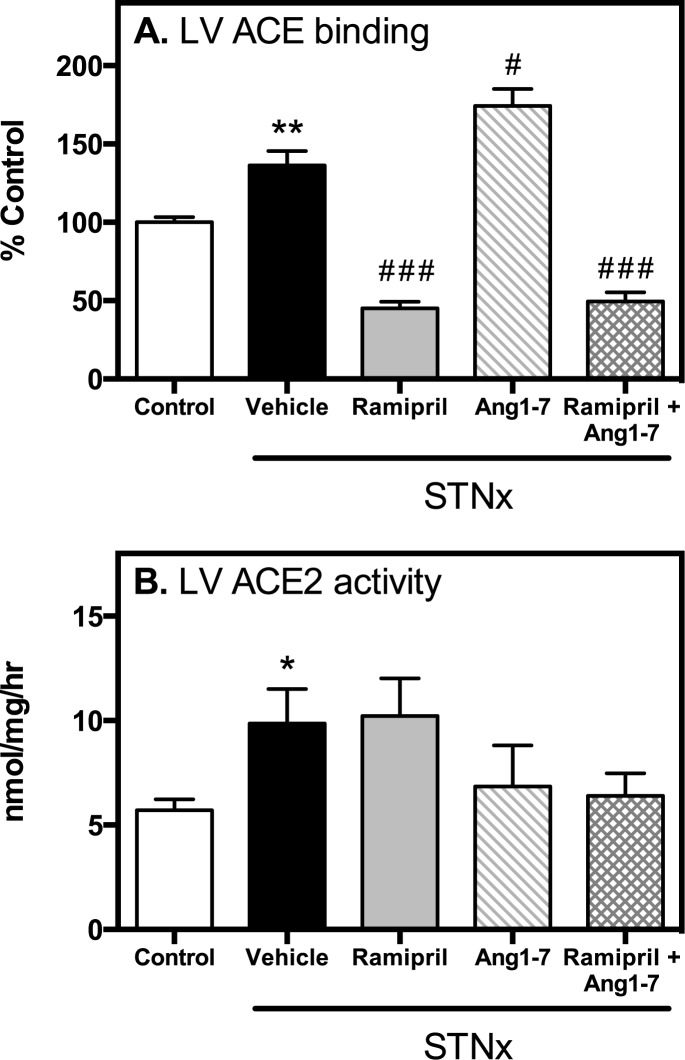
Cardiac ACE and ACE2. Left ventricular (LV) ACE binding (A) and ACE2 activity (B) in control (n = 10) and STNx (vehicle, ramipril, Ang 1–7, ramipril + Ang 1–7; n = 12/group) rats. Data expressed as mean±SEM. *P<0.05, **P<0.01 vs. control and # P<0.05, ### P<0.001 vs. STNx

STNx increased left ventricular ACE (Fig 3A, P<0.01) and ACE2 activity (Fig 3B, P<0.05). Ramipril alone and in combination with Ang 1–7 led to inhibition of cardiac ACE activity (Fig 3A, P<0.001), with no change in cardiac ACE2 activity. The adverse effect of Ang 1–7 on cardiac fibrosis was associated with increased cardiac ACE binding (P<0.001), and non-significant decrease in cardiac ACE2 activity.

## Discussion

The results of the current study confirm our previous report in experimental kidney disease [15] that exogenous infusion of Ang 1–7 has adverse effects to increase blood pressure and accelerate cardiac fibrosis. These changes were associated with increased cardiac ACE. In this study we tested the hypothesis that the addition of an ACE inhibitor to exogenous Ang 1–7 may unmask potential beneficial cardiac effects of Ang 1–7. This hypothesis was not proven, and whilst the addition of ACEi to Ang 1–7 prevented the deleterious actions of Ang 1–7 to increase blood pressure and cardiac fibrosis, no additional benefits of Ang 1–7 were observed using combination therapy.

The results in most experimental models of heart disease have shown a beneficial effect of infusion of Ang 1–7 on the heart [7, 8, 32–34]. However, studies of exogenous Ang 1–7 infusion in models of heart disease secondary to kidney disease have produced conflicting results. A 4 week infusion of Ang 1–7 improved cardiac fibrosis but had no effect on blood pressure in uninephrectomised DOCA salt rats, however renal function was not reported and neither systemic nor tissue RAS components were measured [34]. Initial findings in a mouse model of renal mass reduction showed that a 12 week Ang 1–7 infusion reduced blood pressure and improved cardiac remodelling [35]. The mechanism of the benefit was unclear as both Ang 1–7 and hydralazine lowered blood pressure and improved renal function but only Ang 1–7 improved cardiac hypertrophy and fibrosis [35]. Furthermore, the authors did not measure circulating or cardiac ACE, ACE2 or Ang 1–7. Major differences between the two studies include the species used (rat vs. mouse), the method of induction and chronicity of kidney failure (acute vs. chronic) and the duration of Ang 1–7 infusion (10 days vs. 12 weeks). A study in rats with 5/6 nephrectomy reported that an 8 week Ang 1–7 infusion (24 μg/kg/hr, subcutaneous) reduced blood pressure and ameliorated renal injury in nephrectomised rats [36], but did not report on the cardiovascular effects. We have shown that short-term Ang 1–7 infusion increased blood pressure and had adverse cardiovascular effects in the STNx rat [15], but it remains unknown if the adverse cardiac effects of Ang 1–7 are also seen in chronic renal disease secondary to renal mass reduction in the rat. With regards to the renal effects of Ang 1–7, it has been reported that Ang 1–7 administration had adverse renal effects in a model of unilateral uretaral obstruction (UUO) in the mouse [17] and uninephrectomised sheep [19], was not renoprotective in a rat model of focal segmental glomerulosclerosis [18], had no effect on renal function or hypertension in the Goldblatt model of 2-kidney, 1-clip hypertension (2K1C) [37], and increased mesangial area in a mouse model of 5/6 STNx [38].

The exact mechanism responsible for the adverse cardiac and kidney effects of Ang 1–7 is not completely understood, however it has been suggested that the adverse effects are due to activation of the ACE/Ang II/ AT_1_R pathway. Our previous work [15] and the results of the current study suggest that exogenous increases in Ang 1–7 up-regulate cardiac ACE to promote the breakdown of excess Ang 1–7. This has the unintended consequence of increasing Ang II production, and leading to increased fibrosis and hypertrophy. A limitation of the current study is that we were unable to measure tissue angiotensin peptide levels due to a lack of available tissue, however this hypothesis is supported by a recent study demonstrating that exogenous Ang 1–7 administration increases kidney Ang II levels in a dose dependent manner in the obstructed kidney of UUO mice when compared to vehicle treated rats, with no effect on the unobstructed kidney [17].

Another potential mechanism responsible for the adverse effects of Ang 1–7 could involve the *mas* receptor. Although *mas* knock out (KO) mice have glomerular hyperfiltration and renal fibrosis suggesting a protective role for the *mas* receptor [39], other reports support our own view that Ang 1–7 may have adverse effects in kidney disease via the *mas* receptor. For example, *mas* KO mice have been shown to have reduced renal damage after UUO [16]. We have also reported that Ang 1–7 caused transition of tubulo-epithelial cells into myofibroblasts [tubular epithelial-to-mesenchymal transition (EMT)], an important contributor to renal fibrosis [40]. This effect in a normal rat kidney cell line (NRK-52) was exclusively dependent on the Ang 1-7/*mas* receptor pathway with upregulation of transforming growth factor-beta (TGF-ß), fibronectin and α-smooth muscle actin (αSMA) with Ang 1–7, and reversal with the *mas* receptor antagonist, A779 [40]. The *in vitro* work was supported by *in vivo* data, demonstrating that infusion of Ang 1–7 was associated with increased renal expression of EMT markers [40]. More recently, Alzayadneh et al. reported that a lower dose of Ang 1–7 abolished EMT induced by advanced glycation end products and TGF-ß in NRK-52 cells, effects that were also blocked by *mas* inhibition, suggesting a potential dose effect of the peptide [41].

The action of Ang 1–7 in kidney disease can vary depending on whether the source of Ang 1–7 is endogenous (low dose) or exogenous (high dose). Zimmerman et al [17] elegantly showed in UUO mice that blocking the effects of endogenous Ang 1–7 using the *mas* receptor antagonist, A779 exacerbated injury in the obstructed kidney, and that exogenous Ang 1–7 administration worsened kidney injury and inflammation. These results suggest dose-dependent effects of Ang 1–7 in the kidney in UUO, with endogenous Ang 1–7 being protective via the *mas* receptor, and higher exogenous Ang 1–7 levels exacerbating kidney injury partly via non-*mas* receptor pathways [17]. By contrast, Kim et al [42] reported that Ang 1–7 prevented obstructive nephropathy in UUO rats and suggested that the discrepancies in the two studies may relate to differences in dose and duration of Ang 1–7, or the animal species used. Neither Zimmerman [17] nor Kim [42] reported on the cardiac consequences of Ang 1–7 in their studies.

To date, relatively few studies have assessed the cardiovascular effects of the ACE2/Ang 1-7/*mas* receptor axis in kidney disease. To our knowledge, this is the first study to examine the effects of combining Ang 1–7 therapy with an ACE inhibitor in the STNx rat model of kidney injury. We confirm our previous work [15] that Ang 1–7 has deleterious effects to increase blood pressure and exacerbate cardiac fibrosis in STNx rats in association with increased cardiac ACE activity. Although the addition of an ACEi to Ang 1–7 prevented the adverse cardiovascular effects of Ang 1–7, a limitation of the study is that the large increase in Ang 1–7 concentration with ramipril may have masked any effect of infused Ang 1–7. In conclusion, the results do not support a role for exogenous Ang 1–7 therapy in this model of kidney disease either alone or in combination with standard ACEi therapy. Given the conflicting findings reported with Ang 1–7 in the context of kidney injury, future research should focus on increasing ACE2 activity in combination with ACEi in kidney disease.
